# Onset timing and duration of augmented renal clearance in a mixed intensive care unit

**DOI:** 10.1186/s40560-023-00660-9

**Published:** 2023-03-23

**Authors:** Ryusei Mikami, Mineji Hayakawa, Shungo Imai, Mitsuru Sugawara, Yoh Takekuma

**Affiliations:** 1 Department of Pharmacy, Hokkaido University Hospital, Sapporo, 060-8648 Japan; 2 Department of Emergency Medicine, Hokkaido University Hospital, Sapporo, 060-8648 Japan; 3 Faculty of Pharmacy, Keio University, Tokyo, 105-8512 Japan; 4 Faculty of Pharmaceutical Sciences, Hokkaido University, Sapporo, 060-0812 Japan

**Keywords:** Augmented renal clearance, Urinary creatinine clearance, Critical care, Intensive care unit

## Abstract

**Background:**

Augmented renal clearance (ARC) is associated with lower blood plasma concentrations of renally excreted drugs; however, its time course is unknown. The current study aimed to determine the onset timing/duration of ARC, its risk factors, and its association with clinical outcomes by continuous monitoring of urinary creatinine clearance (CrCl) in critically ill patients.

**Methods:**

Data were retrospectively obtained from the medical records of 2592 critically ill patients admitted to the intensive care unit (ICU) from January 2019 to June 2022 at a tertiary emergency hospital. Among these, patients with continuously measured urinary CrCl were selected and observed over time. We evaluated the onset timing and duration of ARC by plotting Kaplan–Meier curves. Furthermore, by multivariate analyses, factors associated with the onset and persistence of ARC were analyzed, and the association between the ARC time course and clinical outcomes was evaluated.

**Results:**

The prevalence of ARC was 33.4% (245/734). ARC onset was within 3 days of admission in approximately half of the cases, and within 1 week in most of the other cases. In contrast, the persistence duration of ARC varied widely (median, 5 days), and lasted for more than a month in some cases. Multivariate analysis identified younger age, male sex, lower serum creatinine at admission, admission with central nervous system disease, no medical history, use of mechanically assisted ventilation, and vasopressor use as onset factors for ARC. Furthermore, factors associated with ARC persistence such as younger age and higher urinary CrCl on ARC day 1 were detected. The onset of ARC was significantly associated with reduced mortality, but persistent of ARC was significantly associated with fewer ICU-free days.

**Conclusions:**

Despite the early onset of ARC, its duration varied widely and ARC persisted longer in younger patients with higher urinary CrCl. Since the duration of ARC was associated with fewer ICU-free days, it may be necessary to consider a long-term increased-dose regimen of renally excreted drugs beginning early in patients who are predicted to have a persistent ARC.

**Supplementary Information:**

The online version contains supplementary material available at 10.1186/s40560-023-00660-9.

## Background

Augmented renal clearance (ARC) is a phenomenon of increased renal excretion of circulating solutes, mostly defined by creatinine clearance (CrCl) > 130 mL/min/1.73 m^2^ [1, 2]. Since the first report by Udy et al. [3], ARC has gradually gained more attention; however, its importance has not yet been fully recognized in general clinical practice. Physicians and pharmacists are usually less likely to actively suspect ARC, because they often adjust (i.e., reduce) the dosage of renally excreted drugs to account for decreased renal function. However, ARC in critical care settings is not uncommon [4, 5]. This increased renal clearance above the normal range indicates a potential underdosing risk for many renally excreted drugs and contributes to treatment failure [6–8].

Previous ARC studies have reported its developmental mechanisms, prevalence, risk factors, and pharmacokinetics in renally excreted drugs [1–6]. Although there is a paucity of specific data on dosing regimens in patients with ARC, underdosing of antimicrobials is frequently reported and is an important issue in critically ill patients, where it is more likely to affect therapeutic outcomes [7, 9]. Furthermore, to evaluate renal function for drug administration in patients with ARC, direct measurement of urinary CrCl is recommended instead of alternative renal function estimation equations using serum creatinine (SCr), such as the Cockcroft–Gault (C–G) equation [10], modification of diet in renal disease (MDRD) equation [11], and Chronic Kidney Disease Epidemiology Collaboration (CKD-EPI) equation [12], which are commonly used in clinical practice. Although urinary CrCl is inexpensive and practical, it requires labor to store urine. Therefore, few hospitals continuously and directly measure urinary CrCl in all critically ill patients, especially those without decreased renal function, contributing to the lack of recognition of ARC [13]. Despite the increased focus on ARC, a general lack of awareness about ARC and the need for continuous monitoring of urinary CrCl have impeded individualized drug design. Above all, the time course of ARC remains unknown, and the duration and risk factors for transient or persistent ARC symptoms are not clear [6, 14]. In patients with ARC, higher doses than the standard is required for renally excreted drugs; however, the specific duration of administration is unknown. This time-course study is essential, because high-dose regimens of renally excreted drugs for ARC may be excessive after ARC state has ended.

Considering the above, this study aimed to determine the time course of ARC (onset and duration) and to identify its risk factors by continuous direct measurement of urinary CrCl in critically ill patients. As an additional analysis, we examined the association between the time course of ARC and clinical outcomes.

## Methods

### Data source

All data were retrospectively obtained from the medical records of 2592 critically ill patients admitted to the intensive care unit (ICU) from January 2019 to June 2022 at Hokkaido University Hospital (a tertiary care hospital). The urinary CrCl was measured at least once for nearly all of the initially screened patients. Of these, critically ill patients for whom urinary CrCl was continuously measured, were selected and observed over time. Patients were excluded if they were younger than 18 years, received renal replacement therapy, or had measurement deficiencies during the observation period (including discontinuous urinary CrCl measurements and short-term ICU stays within 3 days).

### Renal function evaluation equation

Urinary CrCl was calculated over 6–24 h. The estimated CrCl value was calculated using the C–G equation [10]. The estimated glomerular filtration rate (GFR) was calculated using the MDRD [11] and CKD-EPI [12] equations. In this study, all renal function evaluation equations were represented on a per body surface area basis. We calculated body surface area using the Du-Bois formula [15].

### Definitions and observations

Based on previous studies on non-achievement of target concentrations of renally excreted drugs, the cutoff for ARC was defined as urinary CrCl > 130 mL/min/1.73 m^2^ [1, 2, 6, 16]. In all critically ill patients included in the analysis, urinary CrCl levels were measured daily over time from ICU admission. In some non-ARC patients, we allowed measurement on the next day if urinary CrCl could not be measured but excluded patients if it could not be evaluated for more than 2 days. The maximum observation period for urinary CrCl until the onset of ARC was 30 days. The onset timing and duration for the first observed ARC were recorded.

### Statistical analysis

Continuous data are presented as mean (standard deviation) or median (interquartile range). Categorical data are presented as counts (%).

The onset timing and duration of ARC were evaluated by plotting the cumulative incidence of ARC using Kaplan–Meier (KM) curves. The estimated time to ARC onset and duration of ARC were evaluated as the time from ICU admission to the onset of ARC and the time from the onset of ARC to the end of ARC, respectively. Furthermore, to identify independent factors associated with the onset and persistence of ARC, multivariate Cox regression models were used. The covariates used were based on the ARC consensus in the current literature [1, 2, 17–19] and data availability. The covariates used were age, sex, renal function, sequential organ failure assessment (SOFA) score, diagnosis at ICU admission, medical history, mechanically assisted ventilation, and vasopressor use (detailed description in Additional file 1: Table S1).

In addition, to evaluate the association between the time course of ARC and mortality a multivariate logistic model was used. In a similar fashion, a multivariate Cox regression model was used to evaluate the association between the time course of ARC and fewer ICU-free days. The covariates used were SOFA score at admission and subsequent degree of change, the ARC time course, plus factors selected from the aforementioned analyses based on the number of observed events and availability of data. The covariates used were SOFA score status (SOFA score at admission and subsequent degree of change), ARC status (presence of transient and persistent ARC with non-ARC as a reference), age, diagnosis at ICU admission, mechanically assisted ventilation, and vasopressor use (detailed description in Additional file 1: Table S2). Because no study has reported on the ARC time course to define the cutoff points for transient and persistent ARC, we established the median duration of ARC in KM curves. ICU-free days were defined as 28 days minus the number of days in the ICU (range: 0–28 days). For circumstances in which death occurred within 28 days and/or ICU stay was more than 28 days, ICU-free days were recorded as 0.

Two-sided *p* values < 0.05 were statistically significant. Statistical analyses were performed using JMP version 16.1 statistical software (SAS Institute Inc., Cary, NC, USA).

## Results

**Fig. 1:**
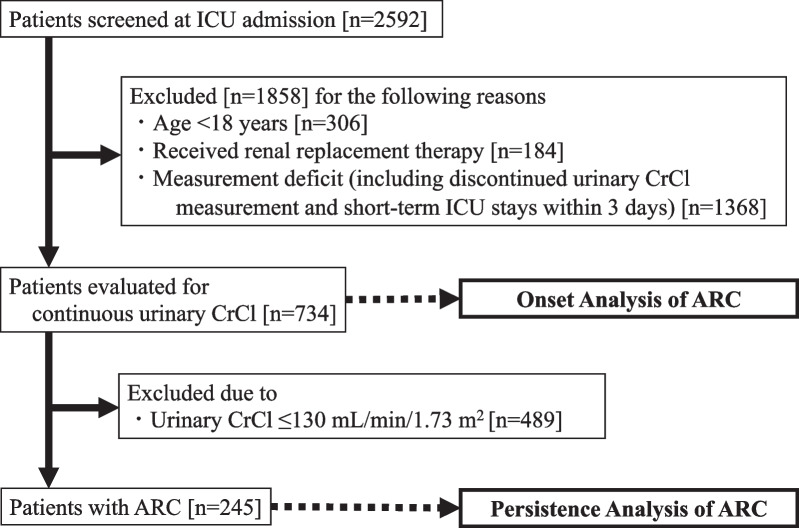
Study flowchart. *ICU* intensive care unit, *CrCl* creatinine clearance, *ARC* augmented renal clearance

A total of 2592 patients were admitted to the ICU during the study period, of whom 734 met the inclusion criteria (Fig. 1). The prevalence of ARC in critically ill patients for whom urinary CrCl was consecutively measured was 33.4% (245/734). The baseline characteristics and laboratory data of the study population were typical of the profile of a mixed ICU cohort (Table 1) [13, 20, 21]. The median age was 70 years (55–79) and 58.2% of the patients were male. In patients with ARC, the median (interquartile range) urinary CrCl at ARC onset was 150.6 (137.2–176.9) mL/min/1.73 m^2^, but the median (interquartile range) C–G equation, MDRD equation, and CKD-EPI equation were 103.6 (86.3–133.5) mL/min/1.73 m^2^, 99.7 (84.1–130.8) mL/min/1.73 m^2^, and 88.7 (79.9–100.5) mL/min/1.73 m^2^, respectively, which were lower than urinary CrCl.

**Table 1:** Patient characteristics (*n* = 734)

	Summary data
Age, years^a^	70 (55–79)
Male sex^b^	427 (58.2%)
Weight, kg^a^	57.1 (47.8–66.9)
Body surface area, m^2a^	1.60 (1.44–1.73)
Serum creatinine, mg/dL^a^	1.04 (0.76–1.46)
Albumin, g/dL^a^	3.2 (2.6–3.7)
Blood urea nitrogen, mg/dL^a^	20 (15–33)
Admission diagnosis^b^	
Trauma	95 (12.9%)
Central nervous system disease^c^	96 (13.1%)
Sepsis	75 (10.2%)
Cardiovascular diseases	278 (37.9%)
Digestive diseases	41 (5.6%)
Infection (without sepsis)	82 (11.2%)
Other	103 (14%)
SOFA score^a^	5 (3–8)
ICU-free days^a^	21 (11–24)
Mortality^b^	103 (14%)

^a^Median (interquartile range)

^b^Number (%)

^c^Central nervous system disease refers to hospitalization for any of the following reasons: traumatic brain injury, intracerebral hemorrhage, subarachnoid hemorrhage, cerebral arteriovenous malformation, hydrocephalus, and status epilepticus

*SOFA* sequential organ failure assessment, *ICU* intensive care unit

### Characteristics of ARC onset

**Fig. 2:**
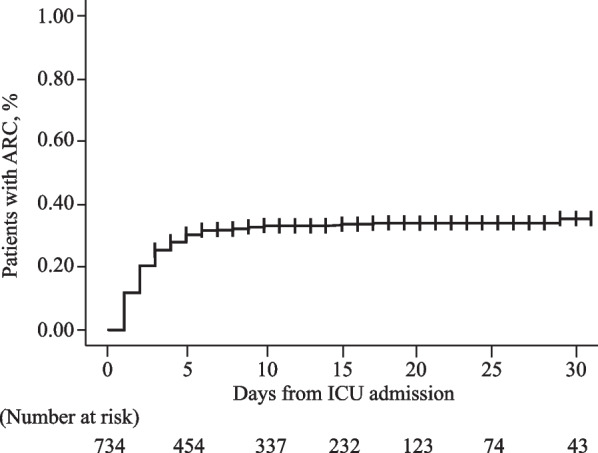
Cumulative incidence rate of ARC (*n* = 734). *ARC* augmented renal clearance, *ICU* intensive care unit

**Fig. 3:**
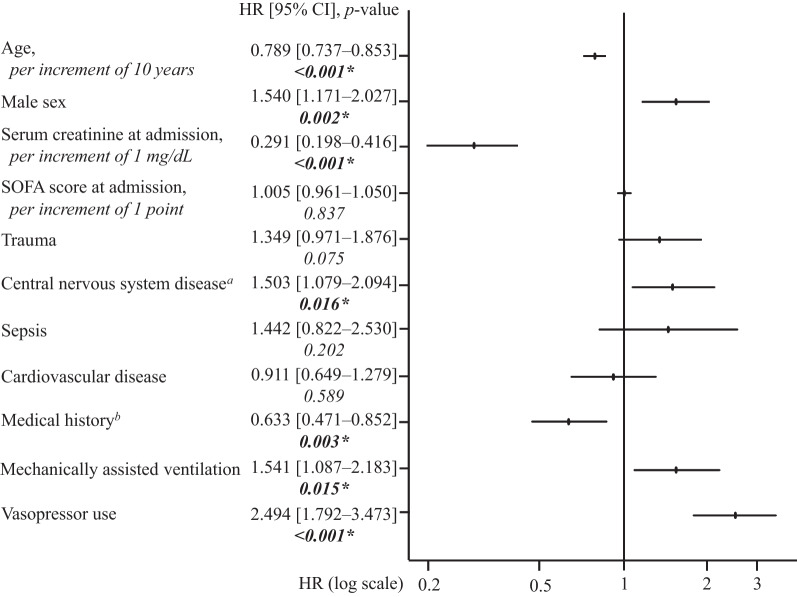
Factors associated with onset of ARC in a mixed ICU population (*n* = 734). ^a^Central nervous system disease refers to hospitalization for any of the following reasons: traumatic brain injury, intracerebral hemorrhage, subarachnoid hemorrhage, cerebral arteriovenous malformation, hydrocephalus, and status epilepticus. ^b^Medical history of any of the following: chronic kidney disease, hypertension, diabetes, myocardial infarction, heart failure, chronic obstructive pulmonary disease, cirrhosis and liver failure. *SOFA* sequential organ failure assessment, *HR* Hazard ratio, *CI* confidence interval. *Significantly different (*p* value < *0.05*)

The median onset time of ARC was undefined, because the KM curve did not cross 50% given the small number of progression events (Fig. 2). In overall patients with ARC, the ARC onset occurred within 3 days of admission in approximately half of the cases, and within 1 week in most of the other cases. Multivariate Cox regression analysis identified younger age, male sex, lower SCr at admission, admission with central nervous system disease, no medical history, use of mechanically assisted ventilation, and vasopressor use as independent factors for development of ARC in a mixed ICU cohort (Fig. 3).

### Duration of ARC

**Fig. 4:**
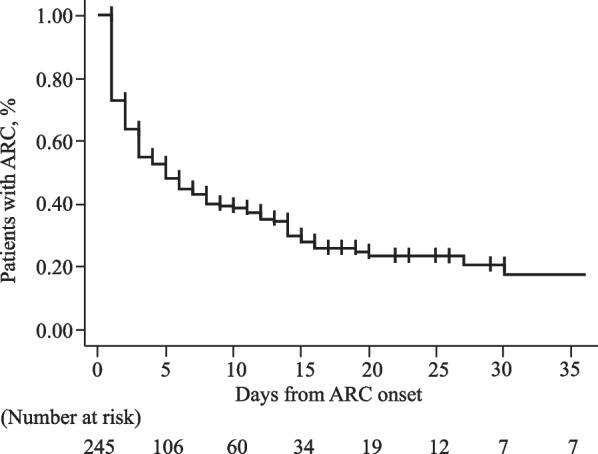
Cumulative persistence rate of ARC (*n* = 245). *ARC* augmented renal clearance

**Fig. 5:**
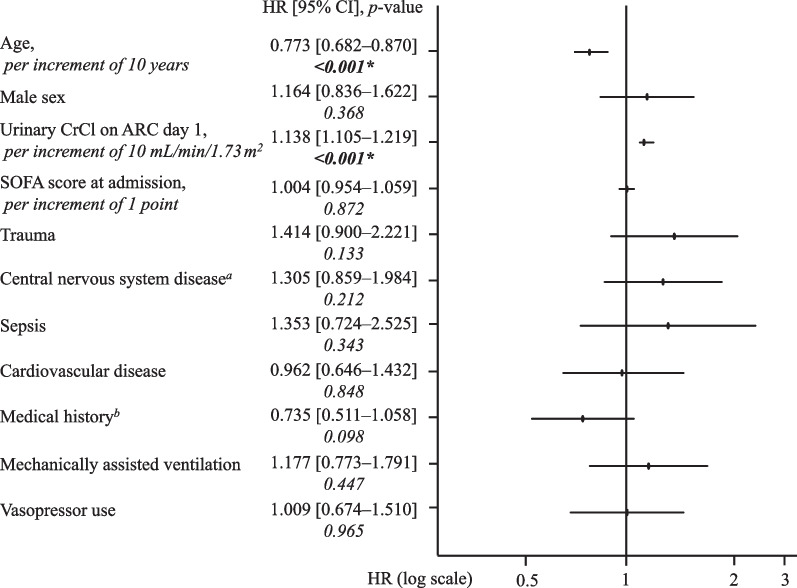
Factors associated with persistence of ARC (*n* = 245). ^a^Central nervous system disease refers to hospitalization for any of the following reasons: traumatic brain injury, intracerebral hemorrhage, subarachnoid hemorrhage, cerebral arteriovenous malformation, hydrocephalus, and status epilepticus. ^b^Medical history of any of the following: chronic kidney disease, hypertension, diabetes, myocardial infarction, heart failure, chronic obstructive pulmonary disease, cirrhosis and liver failure. *ARC* augmented renal clearance, *SOFA* sequential organ failure assessment, *HR* Hazard ratio, *CI* confidence interval. *Significantly different (*p* value < *0.05*)

The median duration of ARC in KM curves was 5 days and ended within 3 weeks in many of those cases (Fig. 4). The duration of ARC had greater variability than the time of onset, with some cases persisting for more than a month. Multivariate Cox regression analysis identified younger age and higher urinary CrCl on ARC day 1 as independent factors for persistent ARC (Fig. 5).

### Relationship between time course of ARC and clinical outcomes

The factors associated with mortality or ICU-free days in a mixed ICU cohort are shown in Additional file 1: Tables S3–S4. The cutoff point for classifying the duration of ARC persistence was set as 5 days, which was the median time in KM curves. The onset of ARC was significantly associated with reduced mortality. In contrast, persistent ARC was significantly associated with fewer ICU-free days.

## Discussion

Although it has been suggested that renal excretory drug doses may need to be adjusted upward in patients with ARC, the specific duration of ARC is unknown [7]; thus, the duration must be clarified to prevent excessive drug exposure. To our knowledge, this is the first study to capture in detail the time course (onset timing and duration) of ARC and its risk factors.

There are few reports on the ARC onset timing, but basically the phenomenon of ARC appears relatively early. Patients with traumatic brain injury have been reported to have an early onset of ARC, with many patients showing markedly elevated renal function parameters in the first 2 days after getting injured [22, 23]. Udy et al. similarly highlighted the occurrence of ARC during the first week after ICU admission [24]. In contrast, in a study of critically ill patients with COVID-19, the onset of ARC was delayed (mean, 28 days) [25]. This delay in the mean onset timing of ARC may be due to the inclusion of the patients without ARC in the analysis. Among the patients with ARC in this study, the onset of ARC occurred early after ICU admission.

The median duration of ARC in KM curves was 5 days, but there was a larger variation than in the time of onset, with ARC lasting for more than a month in some cases. Although no specific duration of ARC has been reported, multivariate analysis suggests that younger patients and those with higher urinary CrCl on ARC day 1 appear to have more persistent ARC. These persistent factors of ARC indicate greater renal functional reserve (RFR). RFR describes the capacity to increase GFR under certain physiological conditions (e.g., pregnancy and solitary kidney) or pathological stimuli (e.g., hypertension, nephrotic syndrome, and polycystic kidney disease) [26]. This hyperfiltration state may occur in patients who are critically ill, young, traumatized, heavily infused, or have excessive cardiac output, all of which are risk factors for ARC [6, 19, 24, 27]. Thus, it is suggested that it may be necessary to consider a long-term increased-dose regimen of renally excreted drugs in patients with ARC and without depressed potential RFR.

Our ARC cohort study revealed that the onset of ARC was significantly associated with a reduction in mortality, but ARC persistence was significantly associated with fewer ICU-free days. This seemingly contradictory result may reflect the conflicting clinical feature of favorable ARC prognosis and the resistance to renal excretion drug therapy [8, 20, 21, 28, 29]. In general, acute kidney injury in the ICU is associated with increased mortality [30]. In contrast, risk factors for ARC are indicators of good renal function. Therefore, ARC itself is associated with a good prognosis [20, 21, 29]. However, previous reports have not examined the persistence of ARC in detail. Theoretically, in patients with ARC, lower blood plasma concentrations of renally excreted drugs will be frequently observed. In other words, the longer the duration of the ARC, the lower the exposure to the drug and the higher the likelihood of clinical failure. Considering the aforementioned report and the results of this study, it is suggested that although the association between ARC and mortality may be mitigated by good prognostic risk factors for ARC onset, persistent ARC may be associated with clinical failures, such as prolonged patient treatment periods. The impact of antimicrobial underdosing on drug resistance and the biological impact of anticonvulsant and anticoagulant underdosing require further investigation in patients with persistent ARC [31–33].

The choice of renal function assessment formula has a significant influence on ARC diagnosis. In various studies, the most common criterion for ARC is CrCl > 130 mL/min/1.73 m^2^, which recommends urinary CrCl to assess its renal function [2, 4, 34]. In contrast, in patients with ARC, the renal function estimation equation (C–G equation, MDRD equation, CKD-EPI equation) does not correlate with urinary CrCl and is estimated lower than it [28, 35]. Similarly in the present study, the alternative renal function estimation equation was estimated to be lower than urinary CrCl. In the future, it will be necessary to develop a new prediction formula that modifies the current renal function estimation equations by capturing the characteristics of renal function over time in patients with ARC, aiming to improve the ability to estimate renal function.

Despite the inability of alternative renal function estimators correctly evaluate ARC using SCr, direct measurement of urinary CrCl is often not performed continuously, especially in patients without decreased renal function because of the effort required to store urine. Therefore, a prediction score of ARC with high accuracy is necessary. Currently, several scoring systems are available for predicting ARC; however, the adaptability of these scores to critically ill patients in mixed ICUs is limited. The ARC score reported by Udy et al. [36] selected three factors (age ≤ 50 years, trauma, and SOFA score ≤ 4); however, in our study, sepsis, trauma, and SOFA score were not selected as a persistent factor for ARC. The ARCTIC score reported by Barletta et al. [14] (SCr < 0.7 mg/dL, male sex, age < 56 years, age 56–75 years) is more user-friendly but applies only to a small population of patients suffering from trauma. In addition, these scores are intended to detect ARC, but they do not consider its persistence. Renal function is dynamic, especially in patients with ARC. The Gijsen et al. score [18] (days from ICU admission, age, sex, SCr, trauma, and cardiac surgery) may be a more useful adjunct tool for predicting ARC persistence, because it predicts daily ARC in a heterogeneous population of critically ill patients.

This study had some limitations. First, our results were not validated prospectively. In the critical care setting, we were able to obtain statistically adequate numbers of patients with ARC, and considering the high heterogeneity of its target patients, the results should be interpreted with caution. Second, the study design may have introduced a selection bias in ARC incidence, because it excluded patients with short-term ICU stays (< 3 days) and patients with measurement deficits (1368 patients). However, in a random-effects meta-analysis, the prevalence (95% confidence interval) of ARC in mixed ICU was 36% (31–41%), which was not different from the prevalence in the present study (245/734 [33.4%]) [4]. Moreover, the objective of this study was to determine the time course of ARC as evaluated by direct measurement of urinary CrCl, and we were able to obtain the sample size necessary for this purpose. Third, we observed the onset of ARC from the time of ICU admission. We could not rule out the possibility that ARC may have developed before that time. In this study, the cumulative incidence of ARCs on the first day of ICU admission was approximately 10%. Previous ARC studies also appear to be replete with reports of ARC occurring from initial observation [24, 29]. Since CrCl generally has a maximum range of about 120 mL/min/1.73 m^2^ [26, 37], patients with ARC are expected to develop ARC after the event occurs (i.e., after ICU admission). Thus, although some patients had ARC at the time of ICU admission, it appears reasonable that ARC would develop relatively early, because the cumulative incidence of ARC is almost maximal within the first week after ICU admission. Fourth, the persistence of ARC was associated with fewer ICU-free days; however, the specific treatment was not examined. In our cohort study, we adjusted for confounding factors (such as severity and initial diagnosis) as much as possible; however, details of therapeutic drugs were not examined, and our findings must be interpreted with caution. Fifth, in a recent study, direct measurement of 6-h urinary CrCl tended to overestimate patients with ARC compared to GFR using iohexol [38]. Nevertheless, the invasive and labor-intensive aspects of daily measurement of GFR with iohexol to capture the time course of ARC are impractical. Direct measurement of urinary CrCl at defined urine collection intervals is an inexpensive and simple method to improve the accuracy of the dynamic assessment of patients with ARC [7, 35, 39]. Finally, this study examined the persistence of first-onset ARC but not recurrent ARC. In fact, some patients in our study exhibited ARC again after ARC paused initially (unpublished data), which may influence the pharmacokinetics of subsequent renally excreted drugs. Therefore, further elucidation of the ARC time course requires an appropriate definition of ARC and proper monitoring of renal function, which should be evaluated in a multicenter prospective study.

## Conclusions

Despite the early onset of ARC, its duration varied widely and ARC persisted longer in younger patients with higher urinary CrCl. Since the duration of ARC was associated with fewer ICU-free days, it may be necessary to consider a long-term increased-dose regimen of renally excreted drugs beginning early in patients who are predicted to have a persistent ARC. The results of this retrospective cohort study will support physicians and pharmacists in determining the drug dosing regimens for patients with ARC.

## Supplementary Information

**Additional file 1: Table S1.** Detailed description of the covariates used for multivariate Cox hazards analysis to identify factors associated with the onset or persistence of ARC. **Table S2.** Detailed description of the covariates used for multivariate analysis to identify factors associated with mortality or fewer ICU-free days. **Table S3.** Independent factors affecting mortality, as determined by a multiple logistic regression analysis. **Table S4.** Independent factors affecting lesser ICU-free days, as determined by a multiple Cox regression analysis.

## Data Availability

The data sets used and/or analyzed during the current study are available from the corresponding author on reasonable request.

## Abbreviations

ARC: Augmented renal clearance
CrCl: Creatinine clearance
SCr: Serum creatinine
C–G: Cockcroft–Gault
MDRD: Modification of diet in renal disease
CKD-EPI: Chronic Kidney Disease Epidemiology Collaboration
ICU: Intensive care unit
GFR: Glomerular filtration rate
SOFA: Sequential organ failure assessment
RFR: Renal function reserve
