# Supplementary material for: Onset timing and duration of augmented renal clearance in a mixed intensive care unit
Source: J Intensive Care. 2023 Mar 23;11:13. doi: 10.1186/s40560-023-00660-9 (PMC10035487; doi:10.1186/s40560-023-00660-9)
Supplement: Supplementary file 1 — Additional file 1: Table S1. Detailed description of the covariates used for multivariate Cox hazards analysis to identify factors associated with the onset or persistence of ARC. Table S2. Detailed description of the covariates used for multivariate analysis to identify factors associated with mortality or fewer ICU-free days. Table S3. Independent factors affecting mortality, as determined by a multiple logistic regression analysis. Table S4. Independent factors affecting lesser ICU-free days, as determined by a multiple Cox regression analysis. [file 40560_2023_660_MOESM1_ESM.docx]

**Additional files**

**Table S1.** Detailed description of the covariates used for multivariate Cox hazards analysis to identify factors associated with the onset or persistence of ARC.

| 1. Age; years |
| --- |
| 1. Male sex; yes or no |
| 1. Renal function; SCr (mg/dL) at admission for onset analysis and urinary CrCl (mL/min) on ARC day 1 for persistence analysis |
| 1. SOFA score at admission; points |
| 1. Diagnosis at ICU admission |
| - 1. Trauma; yes or no |
| - 1. CNS disease: traumatic brain injury, intracerebral hemorrhage, subarachnoid hemorrhage, cerebral arteriovenous malformation, hydrocephalus, and status epilepticus; yes or no |
| - 1. Sepsis; yes or no |
| - 1. Cardiovascular disease; yes or no |
| 1. Medical history: chronic kidney disease, hypertension, diabetes, myocardial infarction, heart failure, chronic obstructive pulmonary disease, cirrhosis and liver failure; yes or no |
| 1. Mechanically assisted ventilation; yes or no |
| 1. Vasopressor use; yes or no |

ARC: augmented renal clearance; SCr: serum creatinine; CrCl: creatinine clearance; SOFA: sequential organ failure assessment; CNS: central nervous system.

**Table S2.** Detailed description of the covariates used for multivariate analysis to identify factors associated with mortality or fewer ICU-free days.

| 1. SOFA score status |
| --- |
| - 1. SOFA score at admission; points |
| - 1. ΔSOFA score: Max SOFA score–SOFA score at admission; points |
| 1. ARC status |
| - 1. No ARC: urinary CrCl ≤130 mL/min/1.73m^2^; yes or no |
| - 1. Transient ARC: urinary CrCl >130 mL/min/1.73m^2^, and for ≤5 days continuously; yes or no |
| - 1. Persistent ARC: urinary CrCl >130 mL/min/1.73m^2^, and for >5 days continuously; yes or no |
| 1. Age; years |
| 1. Diagnosis at ICU admission |
| - 1. Trauma; yes or no |
| - 1. CNS disease: traumatic brain injury, intracerebral hemorrhage, subarachnoid hemorrhage, cerebral arteriovenous malformation, hydrocephalus, and status epilepticus; yes or no |
| - 1. Sepsis; yes or no |
| - 1. Cardiovascular disease; yes or no |
| 1. Mechanically assisted ventilation; yes or no |
| 1. Vasopressor use; yes or no |

ICU: intensive care unit; SOFA: sequential organ failure assessment; ARC: augmented renal clearance; CrCl: creatinine clearance; SOFA: sequential organ failure assessment; CNS: central nervous system.

**Table S3.** Independent factors affecting mortality, as determined by a multiple logistic regression analysis.

|  | Odds ratio (95% confidence interval) | *p*-value |
| --- | --- | --- |
| SOFA score status |  |  |
| SOFA score at admission,  *per increment of 1 point* | 1.175 (1.067–1.294) | *0.001******** |
| ΔSOFA score*^a^*,  *per increment of 1 point* | 1.220 (1.114–1.336) | *<0.001******** |
| ARC status |  |  |
| Non-ARC | Reference | *-* |
| Transient ARC | 0.429 (0.197–0.933) | *0.033******** |
| Persistent ARC | 0.305 (0.099–0.937) | *0.038******** |
| Age,  *per increment of 10 years* | 1.172 (0.980–1.384) | *0.084* |
| Diagnosis at ICU admission |  |  |
| Trauma | 0.811 (0.308–2.140) | *0.672* |
| Central nervous system disease*^b^* | 0.225 (0.099–0.513) | *<0.001******** |
| Sepsis | 0.776 (0.320–1.877) | *0.573* |
| Cardiovascular disease | 1.440 (0.799–2.595) | *0.226* |
| Mechanically assisted ventilation | 5.706 (1.662–19.59) | *0.006******** |
| Vasopressor use | 1.428 (0.702–2.906) | *0.325* |

*^a^* ΔSOFA score: Max SOFA score–SOFA score at admission.

*^b^* Central nervous system disease refers to hospitalization for any of the following reasons: traumatic brain injury, intracerebral hemorrhage, subarachnoid hemorrhage, cerebral arteriovenous malformation, hydrocephalus, and status epilepticus.

SOFA: sequential organ failure assessment; ARC: augmented renal clearance; ICU: intensive care unit.

******* Significantly different (*p*-value *<0.05*).

**Table S4.** Independent factors affecting lesser ICU-free days, as determined by a multiple Cox regression analysis.

|  | Hazard ratio (95% confidence interval) | *p*-value |
| --- | --- | --- |
| SOFA score status |  |  |
| SOFA score at admission,  *per increment of 1 point* | 1.059 (1.028–1.091) | *<0.001******** |
| ΔSOFA score*^a^*,  *per increment of 1 point* | 1.123 (1.087–1.159) | *<0.001******** |
| ARC status |  |  |
| Non-ARC | Reference | *-* |
| Transient ARC | 1.108 (0.893–1.374) | *0.351* |
| Persistent ARC | 1.195 (1.426–2.655) | *<0.001******** |
| Age,  *per increment of 10 years* | 1.094 (1.041–1.161) | *<0.001******** |
| Diagnosis at ICU admission |  |  |
| Trauma | 1.088 (0.846–1.400) | *0.510* |
| Central nervous system disease*^b^* | 1.175 (0.907–1.521) | *0.222* |
| Sepsis | 0.965 (0.728–1.271) | *0.803* |
| Cardiovascular disease | 1.059 (0.876–1.281) | *0.551* |
| Mechanically assisted ventilation | 1.227 (0.997–1.512) | *0.054* |
| Vasopressor use | 1.225 (0.990–1.517) | *0.062* |

*^a^* ΔSOFA score: Max SOFA score–SOFA score at admission.

*^b^* Central nervous system disease refers to hospitalization for any of the following reasons: traumatic brain injury, intracerebral hemorrhage, subarachnoid hemorrhage, cerebral arteriovenous malformation, hydrocephalus, and status epilepticus.

ICU: intensive care unit; SOFA: sequential organ failure assessment; ARC: augmented renal clearance.

******* Significantly different (*p*-value *<0.05*).
